# Data on greenhouse gases emission of fuels in power plants in Malaysia during the year of 1990–2017

**DOI:** 10.1016/j.dib.2020.105440

**Published:** 2020-03-19

**Authors:** Wan Nurdiyana Wan Mansor, Samsuri Abdullah, Che Wan Mohd Noor Che Wan Othman, Mohamad Nor Khasbi Jarkoni, How-Ran Chao, Sheng-Lun Lin

**Affiliations:** a Faculty of Ocean Engineering Technology & Informatics, Universiti Malaysia Terengganu, 21300, Malaysia; b Air Quality and Environment Research Group, Universiti Malaysia Terengganu, 21300, K. Nerus, Malaysia; c Department of Environmental Science and Engineering, National Pingtung University of Science and Technology, Pingtung 912, Taiwan; d Department of Civil Engineering and Geomatics, Cheng Shiu University, Kaohsiung City 83347, Taiwan; e Center for Environmental Toxin and Emerging-Contaminant Research, Cheng Shiu University, Kaohsiung 83347, Taiwan

**Keywords:** Greenhouse gas emission, Malaysia power plants, Energy data of Malaysia, Emission factor

## Abstract

Energy has a significant influence on Malaysia's industry. It is used in electricity generation, refineries, gas processing plants and end-user applications such as transportation, residential, agriculture and fishing. These burning fossil fuel activities produce greenhouse gases (GHG) emissions. This article presents the emissions data of fuel used in power plants in Malaysia during the year of 1990 until 2017. The fuel used in power plants is coal and coke, natural gas, diesel oil and residual fuel oil. The energy data used in power plants were gathered from the Malaysia Energy Information Hub, published by the Malaysian Energy Commission. The GHG emissions data were calculated using the emission factors method. The climate impact of different GHGs in terms of CO_2_-equivalent (CO_2_-e) was also calculated using global warming potentials. The article also presents population data in Malaysia during the year. A correlation between the fuels, GHG emission and the population is also investigated using statistical analysis. The data presented here may facilitate the Malaysian government to identify the source of the pollutants and undertake a climate change mitigation plan.

Specifications Table

**Table:** 

Subject	Environmental Engineering
Specific subject area	Air Pollution
Type of data	Table
	Figure
	Graph
How data were acquired	Raw data was gathered from the Malaysia Energy Commission and the Department of Statistics, Malaysia.
Data format	Processed, raw
Parameters for data collection	Data of fuel input to power stations and Malaysia's population during the year of 1990 until 2017 were considered.
Description of data collection	The data was processed and analysed using the emission factor method by the 2006 IPCC Guidelines for National Greenhouse Inventories. The global warming potential values from the 2014 IPCC Fifth Assessment Report was used in global warming potential calculation. The data was analysed statistically using Microsoft Excel 2013.
Data source location	Malaysia, 4.2105° N, 101.9758° E
Data accessibility	Processed data is available with the article. Direct URL to raw data: https://meih.st.gov.my/publications, http://www.dosm.gov.my and http://www.data.gov.my

## Value of the Data

• These data are useful for climate change mitigation measures in reducing GHG emissions from fossil-fuel-fired power plants.
• Malaysia's government is committed to reducing its greenhouse gas emission under the 2015 Paris Climate agreement by 2030. The government can estimate how much the reduction when switching to renewable energy plants using these data. The data is also beneficial for those who are interested with Malaysia's greenhouse gas emissions trend during the year of 1990 until 2017.
• The data presents the opportunity for greenhouse gas emission reduction in Malaysia from the power plants sector.
• The data shows the trend of Malaysia's populations from 1990 until 2017 and their correlation with the GHG emission from the power plant sector. The data is beneficial for those who are interested in learning the relationship between Malaysia's energy vs. population, either performing the time series or forecasting future trends.

## 1 Data description

**Fig. 1:**
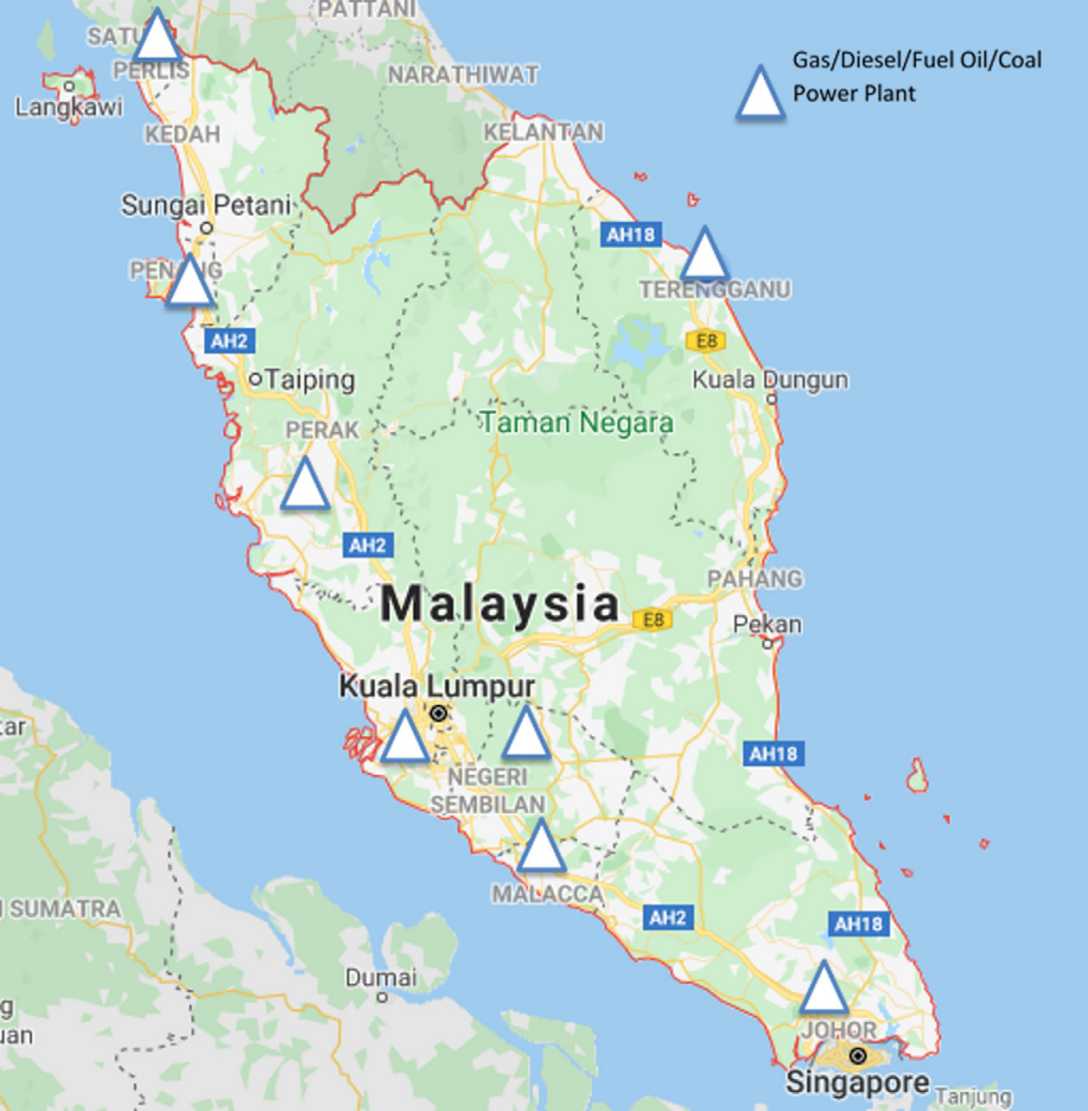
Power plants located in Peninsular Malaysia.

**Fig. 2:**
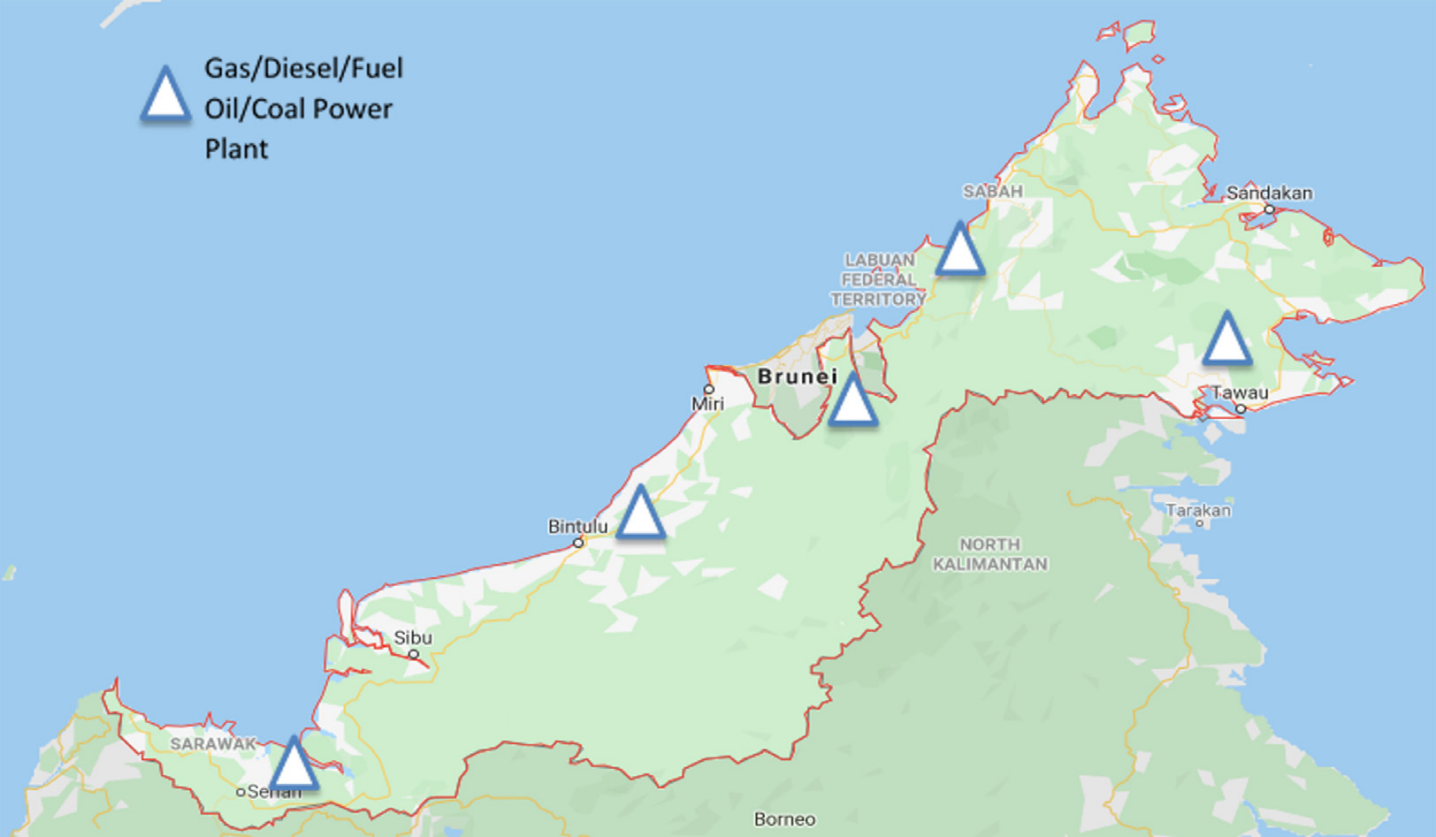
Power plants located in West Malaysia.

**Fig. 3:**
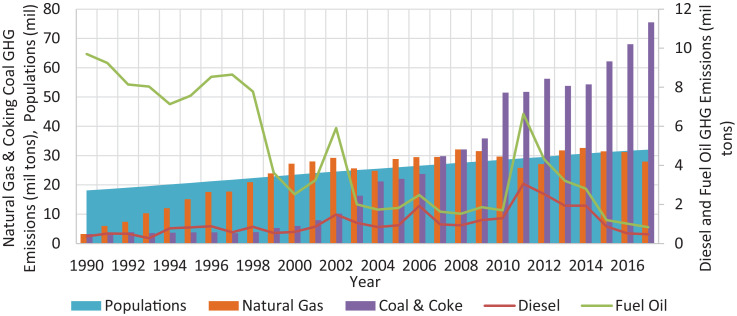
Time-series plot for GHG emissions and population trends.

**Fig. 4:**
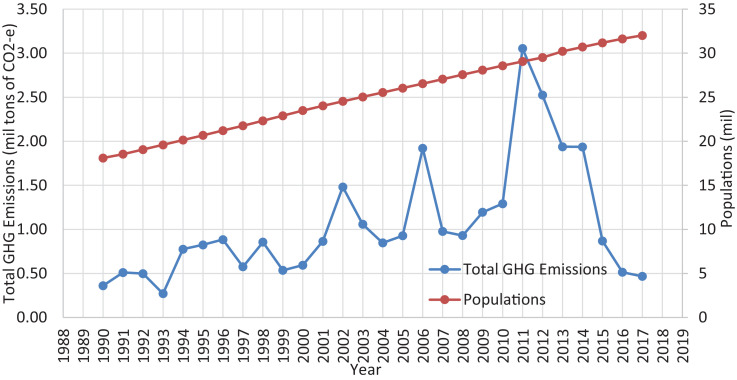
Total GHG emissions in CO_2_-e from power plants and populations in Malaysia.

Data for populations, GHG emissions specifically CO_2_, CH_4_, N_2_O and total emissions in CO_2_-e from fuel power plants in Malaysia during the year of 1990 until 2017 are shown. The total of four figures and seven tables showing each data are presented in this investigation. Fig. 1, Fig. 2 show active power plants in Malaysia using coal, natural gas, diesel and fuel oil in 2018. Table 1 shows the emission factor and global warming potential values for a 100-year limit relative to CO_2_. Table 2 presents the data of Malaysia's populations during the year 1990 until 2017. Table 3 depicts the data of GHG and its total emissions in tons and CO_2_-e, respectively, from natural gas power plants. Table 4, Table 5 and Table 6 present the GHG emissions data in tons and CO_2_-e from diesel, fuel oil and coal and coke power plants, respectively. Fig. 3. illustrates the trend of GHG emissions and populations over time. Fig. 4. represents the total GHG emissions in CO_2_-e from power plants and populations in Malaysia. Table 7 shows the correlation analysis between the total GHG emissions in CO_2_-e from fuels used in power plants and populations.

**Table 1:** Emission factor and global warming potential value.

GHG	Emission Factor (kg/TJ) [4]				GWP values for 100-year time horizon [2]
	Natural gas	Diesel oil	Fuel Oil (as Residual Fuel oil)	Coal and Coke (as coking coal)	Fifth assessment report (AR5)
CO_2_	56,100	74,100	77,400	94,600	1
CH_4_	5	10	10	10	28
N_2_O	0.1	0.6	0.6	1.5	265

**Table 2:** Malaysia's population 1990 – 2017.

Year	Populations	Reference
1990	18,102,400	[5]
1991	18,547,200	[5]
1992	19,067,500	[5]
1993	19,601,500	[5]
1994	20,141,700	[5]
1995	20,681,800	[5]
1996	21,222,600	[5]
1997	21,769,300	[5]
1998	22,333,500	[5]
1999	22,909,500	[5]
2000	23,494,900	[5]
2001	24,030,500	[5]
2002	24,542,500	[5]
2003	25,038,100	[5]
2004	25,541,500	[5]
2005	26,045,500	[5]
2006	26,549,900	[5]
2007	27,058,400	[5]
2008	27,567,600	[5]
2009	28,081,500	[5]
2010	28,588,600	[5]
2011	29,062,000	[5]
2012	29,510,000	[5]
2013	30,213,700	[5]
2014	30,708,500	[6]
2015	31,186,100	[6]
2016	31,633,500	[6]
2017	32,022,600	[6]

**Table 3:** GHG emissions in tons from natural gas power plants in Malaysia, 1990–2017.

Year	GHG emissions (tons)				Annual growth/Reduction
	CO_2_	CH_4_	N_2_O	All GHGs (tons CO_2_-e)	
1990	3196,709.72	56.98	5.70	3,199,815.26	–
1991	5949,497.23	106.05	10.61	5,955,277.04	86.1%
1992	7384,610.85	131.63	13.16	7,391,784.85	24.1%
1993	10,273,628.46	183.13	18.31	10,283,609.07	39.1%
1994	12,023,480.58	214.32	21.43	12,035,161.15	17.0%
1995	15,065,169.85	268.54	26.85	15,079,805.35	25.3%
1996	17,590,124.26	313.55	31.35	17,607,212.70	16.8%
1997	17,688,773.64	315.31	31.53	17,705,957.92	0.6%
1998	20,871,390.59	372.04	37.20	20,891,666.72	18.0%
1999	23,868,452.76	425.46	42.55	23,891,640.47	14.4%
2000	27,199,043.78	484.83	48.48	27,225,467.10	14.0%
2001	28,002,331.61	499.15	49.92	28,029,535.30	3.0%
2002	29,181,426.60	520.17	52.02	29,209,775.75	4.2%
2003	25,585,421.76	456.07	45.61	25,610,277.47	−12.3%
2004	24,768,041.17	441.50	44.15	24,792,102.81	−3.2%
2005	28,822,060.99	513.76	51.38	28,850,061.03	16.4%
2006	29,416,306.08	524.35	52.44	29,444,883.41	2.1%
2007	29,475,025.95	525.40	52.54	29,503,660.33	0.2%
2008	32,063,397.81	571.54	57.15	32,094,546.75	8.8%
2009	31,450,362.37	560.61	56.06	31,480,915.75	−1.9%
2010	29,660,580.73	528.71	52.87	29,689,395.38	−5.7%
2011	25,782,720.52	459.59	45.96	25,807,767.90	−13.1%
2012	27,088,650.43	482.86	48.29	27,114,966.50	5.1%
2013	31,755,705.70	566.06	56.61	31,786,555.71	17.2%
2014	32,554,295.93	580.29	58.03	32,585,921.76	2.5%
2015	31,422,176.83	560.11	56.01	31,452,702.84	−3.5%
2016	31,145,019.05	555.17	55.52	31,175,275.80	−0.9%
2017	27,927,999.30	497.83	49.78	27,955,130.78	−10.3%

**Table 4:** GHG emissions in tons from diesel power plants in Malaysia, 1990–2017.

Year	GHG emissions (tons)				Annual growth/Reduction
	CO_2_	CH_4_	N_2_O	All GHGs (tons CO_2_-e)	
1990	359,880.58	14.57	2.91	361,060.76	–
1991	508,796.68	20.60	4.12	510,465.21	41.4%
1992	496,387.01	20.10	4.02	498,014.84	−2.4%
1993	269,910.44	10.93	2.19	270,795.57	−45.6%
1994	772,502.28	31.28	6.26	775,035.59	186.2%
1995	822,140.98	33.29	6.66	824,837.07	6.4%
1996	881,086.94	35.67	7.13	883,976.33	7.2%
1997	573,947.48	23.24	4.65	575,829.65	−34.9%
1998	853,165.17	34.54	6.91	855,963.00	48.6%
1999	533,616.03	21.60	4.32	535,365.95	−37.5%
2000	592,561.99	23.99	4.80	594,505.21	11.0%
2001	862,472.43	34.92	6.98	865,300.78	45.5%
2002	1476,751.35	59.79	11.96	1,481,594.14	71.2%
2003	1054,822.39	42.71	8.54	1,058,281.53	−28.6%
2004	843,857.91	34.16	6.83	846,625.22	−20.0%
2005	924,520.80	37.43	7.49	927,552.63	9.6%
2006	1914,192.40	77.50	15.50	1,920,469.71	107.0%
2007	974,159.50	39.44	7.89	977,354.12	−49.1%
2008	927,623.22	37.56	7.51	930,665.22	−4.8%
2009	1191,328.82	48.23	9.65	1,195,235.61	28.4%
2010	1287,503.80	52.13	10.43	1,291,725.98	8.1%
2011	3043,472.84	123.22	24.64	3,053,453.46	136.4%
2012	2516,061.65	101.86	20.37	2,524,312.70	−17.3%
2013	1932,806.91	78.25	15.65	1,939,145.27	−23.2%
2014	1929,704.49	78.13	15.63	1,936,032.67	−0.2%
2015	865,574.85	35.04	7.01	868,413.37	−55.1%
2016	511,899.10	20.72	4.14	513,577.80	−40.9%
2017	465,474.51	18.85	3.77	467,000.96	−9.1%

**Table 5:** GHG emissions in tons from fuel oil power plants in Malaysia, 1990–2017.

Year	GHG emissions (tons)				Annual growth/Reduction
	CO_2_	CH_4_	N_2_O	All GHGs (tons CO_2_-e)	
1990	9310,195.53	360.86	72.17	9,339,425.22	–
1991	8707,447.06	337.50	67.50	8,734,784.39	−6.5%
1992	7621,851.69	295.42	59.08	7,645,780.76	−12.5%
1993	7738,512.68	299.94	59.99	7,762,808.01	1.5%
1994	6341,821.32	245.81	49.16	6,361,731.69	−18.0%
1995	6717,728.97	260.38	52.08	6,738,819.52	5.9%
1996	7628,332.85	295.67	59.13	7,652,282.27	13.6%
1997	8043,127.50	311.75	62.35	8,068,379.18	5.4%
1998	6902,442.22	267.54	53.51	6,924,112.67	−14.2%
1999	3078,554.04	119.32	23.86	3,088,219.27	−55.4%
2000	1918,425.25	74.36	14.87	1,924,448.22	−37.7%
2001	2365,625.74	91.69	18.34	2,373,052.70	23.3%
2002	4416,914.90	171.20	34.24	4,430,781.96	86.7%
2003	936,528.54	36.30	7.26	939,468.81	−78.8%
2004	887,919.80	34.42	6.88	890,707.45	−5.2%
2005	891,160.38	34.54	6.91	893,958.21	0.4%
2006	554,139.73	21.48	4.30	555,879.47	−37.8%
2007	644,876.06	25.00	5.00	646,900.67	16.4%
2008	586,545.56	22.73	4.55	588,387.04	−9.0%
2009	664,319.56	25.75	5.15	666,405.21	13.3%
2010	405,072.90	15.70	3.14	406,344.64	−39.0%
2011	3574,363.27	138.54	27.71	3,585,585.11	782.4%
2012	1782,320.76	69.08	13.82	1,787,916.42	−50.1%
2013	1270,308.61	49.24	9.85	1,274,296.79	−28.7%
2014	871,716.88	33.79	6.76	874,453.67	−31.4%
2015	327,298.90	12.69	2.54	328,326.47	−62.5%
2016	502,290.40	19.47	3.89	503,867.35	53.5%
2017	364,653.11	14.13	2.83	365,797.95	−27.4%

**Table 6:** GHG emissions in tons from coal and coke power plants in Malaysia, 1990–2017.

Year	GHG emissions (tons)				Annual growth/Reduction
	CO_2_	CH_4_	N_2_O	All GHGs (tons CO_2_-e)	
1990	3220,059.51	34.04	51.06	3,234,542.97	–
1991	3814,166.43	40.32	60.48	3,831,322.11	18.5%
1992	3833,969.99	40.53	60.79	3,851,214.75	0.5%
1993	3501,270.12	37.01	55.52	3,517,018.43	−8.7%
1994	3663,659.34	38.73	58.09	3,680,138.06	4.6%
1995	3790,402.15	40.07	60.10	3,807,450.95	3.5%
1996	3762,677.16	39.77	59.66	3,779,601.25	−0.7%
1997	3493,348.69	36.93	55.39	3,509,061.37	−7.2%
1998	3818,127.14	40.36	60.54	3,835,300.64	9.3%
1999	5275,669.45	55.77	83.65	5,299,398.81	38.2%
2000	5921,265.64	62.59	93.89	5,947,898.81	12.2%
2001	7897,661.32	83.48	125.23	7,933,184.10	33.4%
2002	10,123,581.92	107.01	160.52	10,169,116.63	28.2%
2003	16,254,765.33	171.83	257.74	16,327,877.41	60.6%
2004	21,098,717.09	223.03	334.55	21,193,616.71	29.8%
2005	21,946,309.62	231.99	347.99	22,045,021.62	4.0%
2006	23,621,691.14	249.70	374.55	23,727,938.81	7.6%
2007	29,649,896.02	313.42	470.14	29,783,257.87	25.5%
2008	31,958,991.58	337.83	506.75	32,102,739.48	7.8%
2009	35,686,022.33	377.23	565.85	35,846,533.98	11.7%
2010	51,295,191.47	542.23	813.35	51,525,911.39	43.7%
2011	51,540,755.67	544.83	817.24	51,772,580.10	0.5%
2012	55,996,557.57	591.93	887.89	56,248,423.69	8.6%
2013	53,576,562.05	566.35	849.52	53,817,543.31	−4.3%
2014	54,055,808.29	571.41	857.12	54,298,945.15	0.9%
2015	61,894,058.93	654.27	981.41	62,172,451.34	14.5%
2016	67,732,149.59	715.98	1073.98	68,036,801.07	9.4%
2017	75,170,368.23	794.61	1191.92	75,508,475.97	11.0%

**Table 7:** Spearman correlation analysis between GHG emissions from fuels and populations.

Fuel type	Natural gas	Diesel	Fuel oil	Coal & Coke
Population	0.876^a^	0.515^b^	−0.867^a^	0.936^a^

a Correlation is significant at the 0.05 level (2-tailed).

b Correlation is significant at the 0.01 level (2-tailed).

## 2 Experimental design, materials, and methods

The energy data used in the research were gathered from the Malaysia Energy Commission [1]. The amount of GHG emitted from power plants was calculated using Eq. (1). For the estimation of the global warming potential from the power generation technologies, the Eq. (2) was used.

(1) E(tons)=A(ktoe)×EF(kg/TJ)

(2) $$\text{Total}\;\text{GHG}\left(\text{tons}\;CO_{2}-e\right)=E\left(\text{tons}\right)\times \text{GWP}\left(CO_{2}-e\right)$$

where *E* is the amount of GHG mass in tons, *A* is the activity data in ktoe and EF is the emission factor in kg/TJ, which is the coefficient established by the Intergovernmental Panel on Climate Change (IPCC) as shown in Table 1 [2]. The total GHG emissions data were converted to CO_2_-e by multiplying the *E* with the global warming potential, GWP values as shown in Table 1. The CO_2_-e reports the equivalent global warming impact from any quantity and type of GHG emissions. The data were processed using an Excel spreadsheet. For the next step in the analysis of the data, the Spearman correlation analysis was applied. The statistical analysis is rated to have a confidence level of 95% [3].

## Appendix Supplementary materials

Image, application 1
